# Regulatory T cells in skeletal muscle repair and regeneration: recent insights

**DOI:** 10.1038/s41419-022-05142-8

**Published:** 2022-08-05

**Authors:** Jianhui Wu, Bowen Ren, Daochao Wang, Hui Lin

**Affiliations:** 1 Cancer Center, Beijing LuHe Hospital, Capital Medical University, Beijing, 101149 China; 2 Jiangxi Province Key Laboratory of Tumor Pathogens and Molecular Pathology and Department of Pathophysiology, School of Basic Medical Sciences, Nanchang University, Nanchang, Jiangxi China; 3 Chinese PLA Medical School, Beijing, 100853 China

**Keywords:** Trauma, Immunotherapy

## Abstract

Skeletal muscle repair and regeneration after injury is a multi-stage process, involving a dynamic inflammatory microenvironment consisting of a complex network formed by the interaction of immune cells and their secreted cytokines. The homeostasis of the inflammatory microenvironment determines whether skeletal muscle repair tissues will ultimately form scar tissue or regenerative tissue. Regulatory T cells (Tregs) regulate homeostasis within the immune system and self-immune tolerance, and play a crucial role in skeletal muscle repair and regeneration. Dysregulated Tregs function leads to abnormal repair. In this review, we discuss the role and mechanisms of Tregs in skeletal muscle repair and regeneration after injury and provide new strategies for Treg immunotherapy in skeletal muscle diseases.

## Facts

- Repair and regeneration after skeletal muscle injury depends on activation and differentiation function of MuSCs.
- Regulatory T cells (Tregs) are key players in skeletal muscle repair and regeneration through immune and non-immune processes.
- Treg cells have a modulatory role in adaptive immunity and direct actions during skeletal muscle repair.

## Open questions

- Skeletal muscle Tregs exhibit pro-repair functions in multiple disease models, but the underlying repair mechanism remains unclear.
- The source of Tregs that exert regenerative effects after skeletal muscle injury is still debated.
- The role of Tregs in skeletal muscle regenerative medicine research and treatment should be further explored.

## Introduction

The skeletal muscle is susceptible to a variety of injuries from internal and external factors during competitive sports and physical exercise. It is subject to a regenerative repair process characterized by a cycle of segmental repair,regeneration and necrosis regardless of whether it is an acute trauma such as mechanical injury, or a chronic degenerative disease such as muscular dystrophy [1]. Although muscle regeneration implies the complete restoration of the original muscle structure, it often causes structural remodeling of the muscle and the formation of scar tissue. Strict regulation of anti-inflammatory responses determines whether skeletal muscle becomes scar tissue or regenerative tissue since excessive anti-inflammatory responses during skeletal muscle repair may lead to pathological fibrosis [2]. Repair and regeneration after skeletal muscle injury is mainly dependent on the activation and differentiation function of muscle stem cells (MuSCs) beneath the basement membrane of the muscle fiber. Immune cells in the inflammatory response secrete cytokines which regulate muscle stem cell proliferation or differentiation and affect the repair process [3–6]. Immune cells may provide potential therapeutic targets for regulating repair and regeneration after skeletal muscle injury. However, few studies have reported the mechanisms of immune cell-mediated skeletal muscle repair and regeneration.

Regulatory T cells (Tregs) are key players in the immune response with immunosuppressive functions helping to regulate some non-immune processes including the functions of other T cells and B cells, and some components of the innate immune system. Tregs participating in muscle repair are activated by the IL-33:ST2 protein axis and recruited to the injury site in large numbers, which promotes the conversion of M1 pro-inflammatory phenotype macrophages to M2 anti-inflammatory phenotype macrophages and releases the specific growth factor amphiregulin (Areg), which stimulates myosatellite cell differentiation and muscle repair and regeneration [7–11]. Therefore, Tregs and their secreted cytokines may serve as new therapeutic targets for healing after skeletal muscle injury, post-fracture repair, and muscular dystrophy. In this review, the role and mechanism of Tregs in the repair and regeneration process after skeletal muscle injury is summarized to provide a valuable reference for skeletal muscle regenerative medicine research and treatment.

## Developmental origins of Tregs

**Fig. 1:**
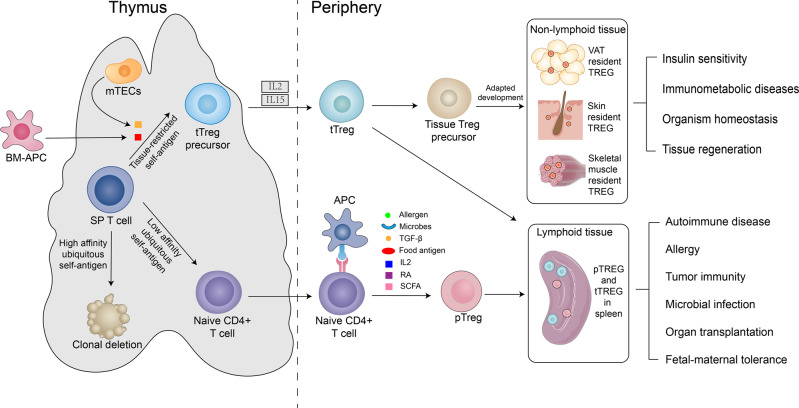
The biological characteristics of Tregs. In the thymus, immature SP cells differentiate into different cell types after binding different functional affinity antigens (TCR signalling) with most SP cells undergoing clonal deletion. Peripheral naive CD4+ Tregs receive signals from low-affinity antigens and are induced by TGF-β, IL-2, RA, and other antigens to differentiate into pTregs. pTregs are mostly found in lymphoid tissues and are mainly responsible for the development of immune tolerance to exogenous antigens. SP cells initially bind to tissue-specific antigens of mTECs or BM-APCs and differentiate into tTregs upon induction of IL-2 and IL-15. Some tTregs are stored in lymphatic tissues, while others are retained in non-lymphoid tissues such as fat, skin, and skeletal muscle through adaptive development where they function in tissue repair, insulin resistance, and maintenance of organismal homeostasis. mTECs, medullary thymic epithelial cells; BM-APCs, bone-marrow derived antigen-presenting cells; SP T cell, single positive T cell; Treg, regulatory T cell; tTreg, thymus-derived Treg; pTreg, peripherally derived Treg; IL-2, interleukin-2; IL-15, interleukin-15; TGFβ1, transforming growth factor beta 1; RA, retinoic acid; SCFA, short chain fatty acid; VAT, visceral adipose tissue.

Tregs are a sub-group of T cells that regulate the autoimmune response and mainly consist of suppressor CD4^+^ T cells [12]. Gershon and Kondo first discovered T cells that suppress the immune response of antigen-specific T cells, however, no surface markers were identified [13]. In 1995, Sakaguchi et al. found that CD4^+^ T cells overexpressing CD25 molecules in mice had immunosuppressive functions and named these cells as CD4^+^ CD25^+^ regulatory T cells [14]. Forkhead/winged helix transcription factor 3 (Foxp3) is the core protein that maintains Treg lineage stability; it plays an important role in the differentiation and immunosuppression of Tregs and in regulating Treg metabolism [15–17]. More importantly, Foxp3 is highly correlated with CD25 overexpression and is a characteristic differentiation marker of Tregs [18–21]. Tregs can be divided into thymus-derived Tregs (tTregs) and peripherally derived Tregs (pTregs) according to site of origin [22]. tTreg differentiates and matures in the thymus and its differentiation is dependent on the strength of T cell receptor (TCR) signalling (functional affinity). TCRs are selected by highly diverse endogenous ligands of the self-peptide-MHC (major histocompatibility complex). In the thymus, immature (CD4^+^CD8^-^) single-positive T cells (SP cells) show different outcomes after receiving different TCR signal intensities by interacting with peptide-MHC on antigen-presenting cells (Fig. 1). SP cells that receive high, moderate, or low intensity TCR signals mostly undergo clonal deletion, escape programmed death and differentiate into Foxp3^+^ Tregs or pTreg in the periphery, respectively. SP cells are transformed into tTreg precursor cells (CD4^+^CD8^−^CD25^+^Foxp3^−^) after presentation of tissue-specific antigens by medullary thymic epithelial cells or bone-marrow derived antigen-presenting cells, and develop into mature tTreg (CD4^+^CD8^−^CD25^+^FOXP3^+^) by the cytokines interleukin-2 (IL-2) and IL-15 [23]. After maturation, tTregs enter the bloodstream and migrate to peripheral lymphoid tissues and lymph nodes, forming a major component of the peripheral Treg pool. In the periphery, naive CD4^+^ T cells are activated and proliferate under the stimulation of antigen-presenting cells, and differentiate and mature under the induction of transforming growth factor β1 (TGF-β1). TGF-β1 is a key factor in the differentiation and maturation of pTregs in vivo and in mediating the conversion of CD4^+^Foxp3^-^ T cells to induced regulatory T cells in vitro [24]. In addition, IL-2, retinoic acid (RA), short chain fatty acid, and external antigens (microbial antigens, food antigens, allergens) contribute to pTreg differentiation [25–28]. Some T cell populations can acquire stable Foxp expression at the inflammation site and at the environmental interface (intestine) and differentiate into pTregs [29–31]. tTregs and pTregs have different T cell receptor pools: the TCR of tTreg is more oriented towards self-recognition and is mainly responsible for immune tolerance to self-antigens, whereas the TCR of pTreg has high affinity for exogenous antigens to generate immune tolerance against non-harmful in vivo substances, and suppresses over-activated pro-inflammatory cells [32–35].

## Functional characteristics of Tregs

Tregs are key regulators of different types of immune responses. For example, they suppress or prevent the immune system from producing inflammatory responses to autoantigens, commensal microbiota, food and environmental antigens [36]. Their immunosuppressive function is exerted by regulating the effector cells of innate and adaptive immunity including macrophages, neutrophils, natural killer cells, dendritic cells, and effector T cells. Specific Foxp3^+^CD4^+^ Treg subpopulations are present in non-lymphoid tissues and human tissues such as skin, muscle, adipose, and colon tissue-resident Treg function in a broader range of disease and biological states [8, 37–39]. It is generally believed that tissue-resident Tregs with little or no expression of the transcription factors Helios or neuropilin originate from the periphery, whereas most tissue-resident Tregs with high Helios and neuropilin expression originate from the thymus [40, 41]. tTregs migrate from the thymus to lymphoid organs, undergo unexplained activation, leave the circulation and enter the tissues, and differentiate into tissue-resident Tregs after undergoing adaptive tissue development. Each tissue-resident Treg population is unique in terms of transcription factor expression, chemokine and adhesion receptor recruitment, and effector function, and all differ from their counterparts in lymphoid organs. Placenta-resident Treg has fetal-maternal tolerance and is a typical peripheral-derived tissue-resident Treg [42]. Treg in adipose tissue is the most characteristic tissue-resident Treg which suppresses adipose tissue inflammation and maintains insulin sensitivity, and is closely associated with immunometabolic disease development [43–46]. The role of skin-resident Treg is to suppress autoimmune and overactive inflammatory responses [47]. In addition, tissue-resident Tregs have repair functions, induce regeneration of parenchymal cells, and play an important role in maintaining homeostasis of the organism [48, 49]. Burzyn showed that skeletal muscle-resident Tregs have highly expressing Helios and neuropilin during acute injury which promotes skeletal muscle repair [7, 8]. In addition, accumulation of skeletal muscle-resident Treg contributes to delaying muscular dystrophy progression in *mdx* (X-linked muscular dystrophy) mice. Treg-induced differentiation in vitro improves the ability of MuSCs to proliferate and differentiate while inhibiting the differentiation of myogenic cells [50]. Although skeletal muscle Tregs exhibits pro-repair functions in multiple disease models, its repair mechanism remains unclear.

## Skeletal muscle injury repair and regeneration

**Fig. 2:**
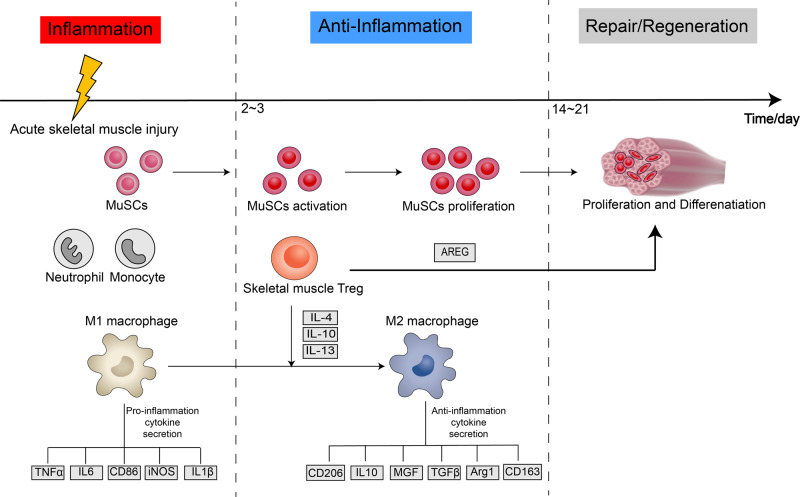
Schematic illustration of the multi-stage of skeletal muscle repair and regeneration. In the early stage of acute skeletal muscle injury, neutrophils, monocytes, and M1 macrophages secrete various pro-inflammatory cytokines to promote the inflammatory response. Within 2–3 days after injury, the inflammatory response state gradually changes to an anti-inflammatory state, MuSCs are activated and begin to proliferate, and Tregs promote the conversion of pro-inflammatory macrophages to anti-inflammatory macrophages and secrete various anti-inflammatory cytokines. Tissue remodeling begins two or three weeks post-injury,and MuSCs proliferate, differentiate, migrate, and fuse to form new muscle fibers; Tregs promote the proliferation of satellite cells, and most immune cells gradually withdraw from the injury site to complete the skeletal muscle repair process. MuSCs, muscle stem cells; TNF-α, tumour necrosis factor-alpha; IL-6, interleukin-6; CD86, cluster of differentiation 86; IL-1β, interleukin-1β; iNOS, inducible nitric oxide synthase; CD206, cluster of differentiation 206; IL-10, interleukin-10; MGF, mechanical growth factor; TGF-β, transforming growth factor-beta; Arg1, arginase1; CD163, cluster of differentiation 163.

Skeletal muscle injuries are common clinical injury disorders, which can be divided into acute injuries (clinically common as mechanical injuries, such as fractures, lacerations and contusions) and chronic injuries (such as ischemic injuries and muscle atrophy diseases). In acute injury, the integrity of the muscle membrane is destroyed and Ca2+ flows in an unregulated manner, activating proteases and phospholipases and generating free radicals to mediate inflammation. This phase is followed by an anti-inflammatory repair phase and a tissue remodeling phase, finally completes the successful repair and regeneration of the injured tissue; the whole process takes approximately two weeks [51, 52]. Therefore, the repair and regeneration process can be divided into three phases: inflammatory injury, anti-inflammatory repair, and tissue remodeling (Fig. 2) [53]. The first 24–48 h after injury results in structural destruction, degeneration, and necrosis of muscle fibers, followed by invading pathogens, necrotic debris, coagulation reactions, and tissue-resident immune cells triggering an inflammatory response leading to the recruitment of various immune cells. Neutrophil-based immune cells are rapidly recruited to infiltrate the injury site for host defense and phagocytosis of necrotic tissue [54]. The number of neutrophils increases after 1 h of injury, secreting chemokines that induce monocytes to migrate to the damaged site and promote the inflammatory response [55]. Pro-inflammatory M1-type macrophages are responsible for the removal of apoptotic leukocytes or necrotic muscle fibers and derived debris, and secrete various pro-inflammatory cytokines, including tumour necrosis factor α, IL-1β, IL-6, cluster of differentiation 86 (CD86), and inducible nitric oxide synthase (iNOS) [56–60]. The pro-inflammatory response gradually begins to become suppressed at 2–3 days after injury by anti-inflammatory macrophages resulting in changes to the anti-inflammatory response. Anti-inflammatory macrophages secrete mechanical growth factor, anti-inflammatory factor IL-10, pro-fibrotic factor TGF-β, arginase 1, cluster of differentiation 163, CD163, and mannose receptor CD206 which promotes injured skeletal muscle repair and regeneration [61–64]. Resting satellite cells become activated, proliferate, and differentiate to form new myotubes that fuse toward surviving muscle fibers and repair damaged muscle fibers. The transition of myeloid infiltration from a pro-inflammatory to an anti-inflammatory state is crucial for skeletal muscle injury repair. A moderate inflammatory response facilitates repair after skeletal muscle injury, whereas excessive inflammation caused by immune cells leads to pathological fibrosis, diminishes tissue function, and may lead to organ failure. Immune cells play an important role in stimulating angiogenesis, myofibroblast activation, and tissue progenitor cell proliferation, with anti-inflammatory M2-type macrophages promoting matrix remodeling and angiogenesis [65, 66]. Repair and regeneration after skeletal muscle injury is a highly coordinated cellular process whether derived from mechanical injury, muscle dystrophy, or infection, and the repair process is largely dependent on the activation and differentiation function of MuSCs [4]. Tissue remodeling begins two to three weeks post-injury; MuSCs are activated, proliferate, differentiate, migrate, and fuse to form new muscle fibers through sequential activation and repression of specific transcription factors, including paired box 7, MyoD, myogenin, and myocyte enhancer factor-2c [67–69]. Most immune cells exit the injury site or are eliminated by apoptosis and tissue homeostasis is restored. Chronic muscle injury leads to similar changes in the cell population of the innate immune system [70]. Muscle membrane damage occurs more rapidly due to weaker muscle membranes, leading unregulated translocation of ions and molecules across the cell membrane. Therefore, many early pathological stages of myotonic diseases are similar to acute muscle injury [71]. The myeloid cells that initially invade mdx muscle at the onset of inflammation are mainly neutrophils and phagocytes, M1 macrophages, and locally expressed iNOS. Similar to acute skeletal muscle injury, neutrophils and M1 macrophages amplify mdx muscle injury by metabolizing iNOS to produce arginine and thus cytotoxic NO. However, unlike acute injury, the recruitment of neutrophils and M1 macrophages to the damaged site is accompanied by the invasion of M2 (M2a type) macrophages, resulting in elevated expression of IL-4, CD206, and arginase, which compete with iNOS for the same substrate, thereby reducing NO production and mitigating the local tissue inflammatory response in the injured muscle [72]. It has been demonstrated that IL-4 treated macrophages can promote their conversion from M1 to M2c phenotype for tissue repair [73]. M2c macrophages produce IL-10 to inactivate M1, while IL-10 can in turn act on macrophages to promote CD163 expression and increase their phagocytosis. IL-10 further promotes the expression of cytokine TGFβ released by T helper type 2 (Th2), regulates muscle stem cell proliferation and differentiation, increases connective tissue produced by fibroblasts, and promotes wound repair. During the acute inflammatory episode dominated by Th1 inflammatory response to the Th2 anti-inflammatory repair phase dominated by M2a and M2c, there is excessive accumulation of connective tissue, which may eventually have serious pathophysiological effects [74].

## Mechanism of Tregs action in skeletal muscle repair and regeneration

Regulatory Tregs have been shown to promote tissue regeneration in acute or chronic tissue injury in the skin, lung, heart, gut and peripheral blood vessels and central nervous system [21, 48, 75–79]. In recent years, the role of T cells, and especially Tregs, in the regeneration and repair of skeletal muscle tissue has received increasing attention. The immune system is extensively involved in the regenerative healing process of skeletal muscle, and immune cells in the inflammatory response and their secreted cytokines regulate the proliferation or differentiation of MuSCs and influence the repair process after skeletal muscle injury [3, 56]. A mouse model of skeletal muscle injury showed that the distinct subpopulations expressing Foxp3^+^CD4^+^ regulatory Tregs significantly increases during acute injury [8]. Meanwhile, muscle injury repair is significantly diminished after reducing their numbers indicating that this class of Tregs plays an important role in the repair of acutely or chronically injured skeletal muscle [8]. In addition, accumulation of Tregs with a similar transcriptome is observed in the muscles of *mdx* mutant mice and patients with myotonic dystrophy disease [50, 80]. Therefore, skeletal muscle Treg, a specialized Treg cell population, may promote the repair process after skeletal muscle injury through a combination of immune and non-immune processes.

### Regulatory T cells control tissue-resident myeloid populations in skeletal muscle repair

After skeletal muscle injury, the early inflammatory response is a necessary element in the repair process. Myeloid cells such as neutrophils and macrophages act as an intrinsic immune barrier, assisting in the removal of surrounding necrotic cells and debris, while promoting the proliferation of parenchymal precursor cells. After skeletal muscle injury, neutrophils release hypochlorite, NADPH oxidase and other cytokines that impair the recovery of skeletal muscle structure and function, while Treg cells can promote skeletal muscle repair by regulating neutrophil behavior and activity [81]. In vitro studies have shown that activated Treg cells promote the secretion of a series of anti-inflammatory factors such as IL-10, TGF-β, haem oxygenase-1 and indoleamine 2,3-dioxygenase by neutrophils recruited at the site of injury, thereby regulating the inflammatory response during the repair process. In addition, Treg cells not only regulate the degree of neutrophil infiltration into damaged areas of skeletal muscle but have also been shown to induce apoptosis of neutrophils, which is essential for skeletal muscle repair. Macrophages play an important role in skeletal muscle repair, and myoglobin released from damaged muscle fibers produces unstable haemoglobin which Tregs and macrophages may recognize as contributing to muscle repair and regeneration regulation [82]. Macrophage deficiency and impaired recruitment leads to impaired muscle repair and regeneration and severe fibrosis at the site of injury [83–86]. Macrophages can secrete cytokines such as tumour necrosis factor-α, IL-1β, and growth differentiation factor 3 (GDF3) to initiate inflammatory responses and promote muscle repair and regeneration [87]. Pro-inflammatory factors accumulate early in skeletal muscle injury, induce myogenic transcription factor expression, and promote apoptosis of fiber/adipose progenitor cells. Alteration of the macrophage phenotype results in anti-inflammatory macrophages producing TGF-β which inhibits fiber/adipose progenitor cell apoptosis and causes cellular matrix deposition in regenerating muscles [88]. Macrophage accumulation and phenotypic changes are central to skeletal muscle repair, and Tregs are essential for maintaining a stable ratio of macrophage subpopulations [89]. Initially, Tregs accumulate in large numbers in damaged tissues and contribute to skeletal muscle repair by regulating the ratio between macrophage subsets and stabilizing the phenotype of macrophages. Tregs are the “switch” that induces the polarization of M1 pro-inflammatory macrophages to M2 anti-inflammatory macrophages by secreting IL-10, IL-4, and IL-13, thus promoting the proliferation and differentiation of MuSCs and muscle repair and regeneration [9, 90]. Meanwhile, two subpopulations of macrophages were identified during muscle repair and regeneration based on the expression of major histocompatibility complex class II (MHCII) molecules [89]. The MHCII**–**macrophage subpopulation is mainly involved in dynamic homeostatic functions, including metabolism and degradation. MHCII+ presents antigens and expresses chemokines and is often in contact with other immune cells. Mice injected with human diphtheria toxin were depleted of Tregs, and MHCII**–**macrophages declined to model cardiotoxin-induced acute skeletal muscle injury [89]. Subsequently, pulsed treatment with 5-ethynyl-2’-deoxyuridine administered to Treg-depleted and Treg-replete mice on the seventh day after injury showed that Treg depletion increased MHCII**–**macrophage death, accelerated MHCII+ macrophages proliferation, enhanced response to interferon-γ (IFN-γ), and elevated pro-inflammatory processes [89]. It is important to note that too strong or too weak an inflammatory response can interfere with the regulation of immune cells during the repair process, which is detrimental to tissue repair. The production of IL-10, IL-4 and IL-13 by Treg cells appears to be a major effector mechanism to control the early inflammatory response. However, there are only speculations based on the enhancement of cytokine expression and too few studies involving the regulatory mechanisms of Treg cells.

### Modulatory role of regulatory T cells in adaptive immunity in skeletal muscle repair

Adaptive immune cells such as Th1, Th2, Th17, and CD8+ T, ensue after skeletal muscle injury. Early in muscle injury, Treg downregulates co-stimulatory molecules CD80 and CD86 in dendritic cells (DCs) in a contact-dependent manner through the expression of inhibitory receptors such as cytotoxic T lymphocyte-associated antigen-4, lymphocyte activation gene-3 and promotes the production of indoleamine 2,3-dioxygenase in DCs [90]. This enzyme limits inflammation triggered by the proliferation of effector T cells, thus promoting the repair of skeletal muscle damage. In addition, Treg not only directly suppresses CD4+ effector T cells by inhibiting co-stimulatory molecules Herpes virus entry mediator /B- and T-lymphocyte attenuator and cytotoxic T lymphocyte-associated antigen-4/B7 but also regulates the skeletal muscle repair process by suppressing IFN-γ produced by Th1 and CD8+ T cells [2, 91, 92]. CD8+ T cells and Th1 produce IFN-γ which activates the CIITA transcription factor and leads to MHCII+ upregulation in macrophages [93]. In contrast, Tregs accumulate early after muscle injury (repair phase) and help control late inflammation by preventing the conversion of MHCII– macrophages to MHCII+ macrophages and limiting the production of IFN-γ by effector T cells to promote muscle repair and regeneration. However, the Treg pathway controlling effector T cells to influence the tissue repair process, and the regulatory mechanism of its Treg–effector T cells–macrophage loop requires in-depth investigation to provide new approaches for immunotherapy after skeletal muscle injury.

### Direct repair of regulatory T cells in skeletal muscle injury

The pro-regenerative effects of regulatory T cells are complex and diverse, and they can act directly or indirectly on non-lymphoid cells or their precursor cells, or even on tissues to promote repair. Tregs act directly on parenchymal cells to promote tissue repair and skeletal muscle regeneration through the epidermal growth factor receptor pathway by secreting Areg. This ligand binds to epidermal growth factor receptor which activates MuSCs. Therefore, Treg acts directly on MuSCs by secreting large amounts of Areg to promote muscle repair by enhancing immune response suppression [11]. Burzyn et al. found better myogenicity in Areg-treated myogenic cells and speculated that it may function to promote muscle repair and regeneration through myogenic differentiation [8, 94]. The mouse model of frozen muscle injury shows a significant increase in the proportion of Tregs on day 4 after cryoinjury, while the high expression of repair-related factors including Areg, IL-10, and TGF-β suggests that they play main roles in promoting muscle regeneration and repair [95]. The role of Treg-derived Areg in promoting tissue repair is demonstrated in a mouse model of influenza virus infection where deletion of Areg expression in Tregs shows a normal antiviral T cell response yet results in severe acute lung tissue injury and decreased blood oxygen concentration [96]. Areg treatment (topical combined with intraperitoneal injection) of mice significantly improves muscle physiological function and immune indices, resulting in decreased expression of T-bet transcription factor in Tregs from muscle. This regulates T cell differentiation toward Th1 cells which inhibits Th1 cell-mediated inflammation to facilitate the reparative effects of CD206hiLy6clo macrophages [97]. Although Areg enhances the ability of Tregs to suppress immunity, it has no effect on the overall proliferation and survival of Tregs. Meanwhile, it affects posttranslational regulation of Foxp3 expression, leading to Foxp3 degradation through epidermal growth factor receptor /GSK-3β signalling in Tregs [98, 99]. In addition, Areg treatment increases the expression of regulatory factors including paired box 7, myogenic factor and myogenin related to muscle stem cell proliferation, myotube activation, and muscle differentiation at the injury site [97].

### Modulation mechanisms of regulatory T cells in skeletal muscle repair

**Fig. 3:**
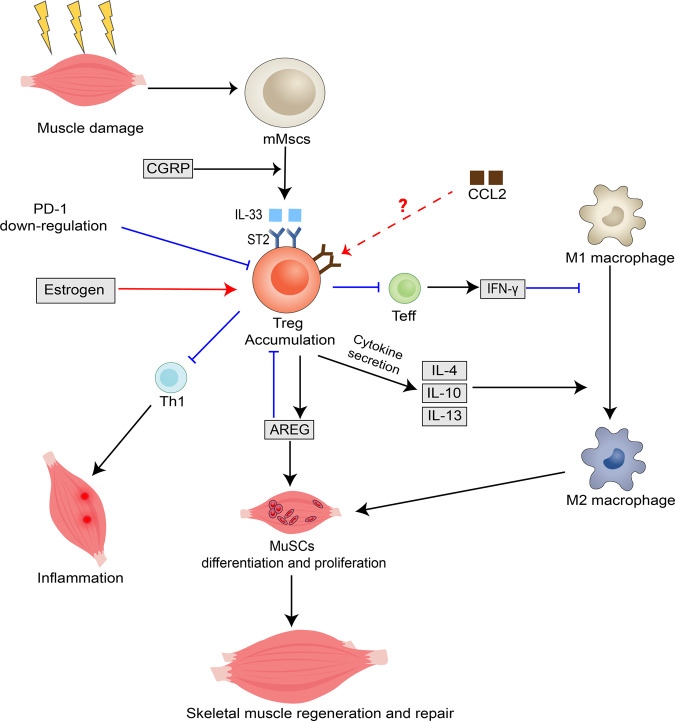
The underlying mechanism of Treg in skeletal muscle repair and regeneration. After muscle injury, mMSCs secrete IL-33 which binds to ST receptors on Tregs causing Treg aggregation at the damaged site. IFN-γ produced by effector T cells causes polarization of pro-inflammatory M1 macrophages to anti-inflammatory M2 macrophages and promotes the proliferation of satellite cells to facilitate tissue repair. Tregs also secrete Areg to influence the repair process by directly promoting the proliferation of satellite cells through the EGFR pathway. Areg inhibits Treg-regulated T cell differentiation to Th1 cells, thereby suppressing the local inflammatory response. Estrogen inhibits Th1 responses in damaged muscle by promoting Treg cell aggregation, thereby reducing inflammation in skeletal muscle. PD-1 downregulation affects Treg aggregation in skeletal muscle and impairs skeletal muscle regeneration, whereas CCL2 may potentially regulate Treg aggregation to affect the repair process. mMscs, muscle mesenchymal stem cells; CGRP, calcitonin gene related peptide; CCL2, chemokine ligand 2; IFN-γ, interferon-γ; Teff, effector T cell; NK cell, natural killer cell; Th1, T-helper 1 cell; EGFR, epidermal growth factor receptor; PD-1, programmed cell death ligand 1.

The mechanism by which Tregs regulate skeletal muscle repair are not fully investigated (Fig. 3). The IL-33:ST2 axis is critical for massive Treg accumulation in skeletal muscle after injury [100, 101]. IL-33 is an endogenous danger signal that responds to tissue injury and promotes recovery from central nerve system injury and Treg aggregation in non-lymphoid tissues, while the ST2 receptor is encoded by the interleukin 1 receptor-like 1 gene. Tregs isolated from injured muscles highly express ST receptors and significantly upregulate the interleukin 1 receptor-like 1 gene compared with Tregs from lymphoid tissues [7, 8, 21]. However, Treg accumulation is impaired in ST2 gene knockout mice, and muscle injury has delayed or impaired recovery [7]. This demonstrates the importance of Tregs and ST2 expression in muscle regeneration after skeletal muscle injury repair [7]. IL-33:ST2 axis drives a positive feedback loop during Treg activation and participates in Treg regulation. IL-33 directly or indirectly enhances the expression and function of Foxp3, GATA binding protein, signal transducer and activator of transcription 5, promotes ST2 protein in Treg expression, promotes Treg aggregation in damaged muscle, and leads to macrophages transforming into the M2 type to repair the damage [102]. IL-33^+^ muscle mesenchymal stem cells crosstalk with nerves and stroma and transcribe a series of genes encoding neuropeptides, neuropeptide receptors, and other neural-related proteins that increase or decrease IL-33 production through up- or down-regulation of calcitonin gene related peptide (CGRP) signalling which affects Treg accumulation in skeletal muscle cells [103]. IL-33 locally promotes the expansion of Tregs in muscle tissue but does not replenish Tregs from circulating blood into muscle tissue [7]. Similarly, intraperitoneal injection of CGRP in mice elevates IL-33 expression, and G2M gene expression promotes Treg differentiation in skeletal muscle 8 h after the injection enriched cell cycle, but not in the spleen, which corroborates its localized effect [103]. In addition, Programmed cell death protein (PD-1) affects skeletal muscle repair since it is essential for the production and development of pTreg [104, 105]. Downregulation of PD-1 gene reduces Treg aggregation and impairs skeletal muscle repair and regeneration in the early stage of injury. Although the number of Tregs increases over time after skeletal muscle injury in PD-1 knockout mice, the expression of Areg secreted by Tregs does not significantly change, suggesting that PD-1 may affect the function of Tregs. It has also been reported that Treg aggregation and function are dependent on estrogen levels, and that estrogen inhibits Th1 responses in damaged muscle by promoting Treg cell aggregation, thereby reducing the inflammatory response in skeletal muscle [106, 107]. Burzyn et al. suggested that muscle Tregs express significantly higher chemokine receptor 2 than Tregs in the spleen suggesting that chemokine ligand 2 may be involved in regulating Treg aggregation in damaged muscle [8]. TCR signalling plays a critical role in regulating Treg differentiation, homeostasis, and function. A small fraction of amphiregulin+ Tregs in the spleen share TCR sequences with amphiregulin+ Tregs or amphiregulin– Tregs in muscle, suggesting that TCR signalling recruits Tregs to accumulate at the injury site [96, 101, 108]. However, the mechanisms by which these signals regulate skeletal muscle repair require confirmation, and further research is needed to identify the signalling pathways involved in influencing Treg aggregation and repair.

## Potential therapeutic targets in Tregs–mediated skeletal muscle diseases- Duchenne muscular dystrophy

**Fig. 4:**
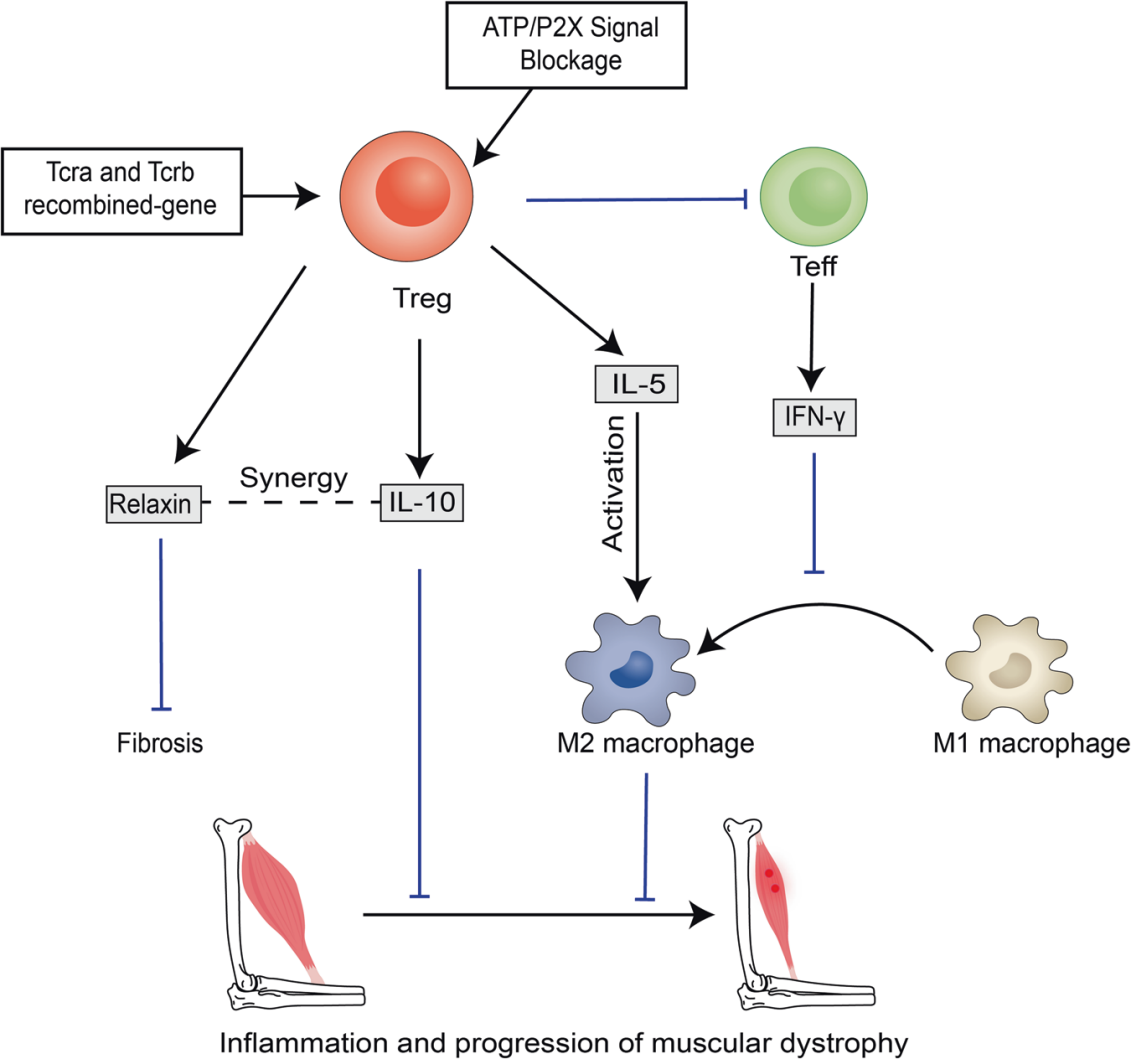
Potential therapeutic targets in Treg mediated muscular dystrophy. Tregs delay the progression of DMD by releasing IL-10. In addition, Tregs suppress the inflammatory response by limiting the release of IFN-γ from effector T cells and releasing IL-5 which promotes the conversion of M1 macrophages to M2 macrophages. Tcra and Tcrb recombinant gene therapy and blockade of the ATP/P2X signalling pathway have potential in DMD treatment by regulating Tregs and delaying muscular dystrophy progression. DMD, Duchenne muscular dystrophy.

Duchenne muscular dystrophy is a fatal degenerative muscle disease caused by loss-of-function mutations in the dystrophin gene [109]. Chronic muscle fiber loss in muscular dystrophy disease is due to a genetic defect wherein satellite cells are repeatedly convened, resulting in their depletion or loss of function over time causing inhibition of the repair process [110]. Treg controls the progression of myotonic dystrophy by suppressing the type I inflammatory response in muscle [80]. Treg accumulation in the skeletal muscle of *mdx* mice helps alleviate inflammation and muscle damage, while Treg depletion exacerbates the peripheral inflammatory response and retards muscle repair [56]. IFN-γ is chronically overexpressed in dystrophic muscle and causes muscle fiber damage by increasing the ratio of M1/M2 macrophages [111, 112]. Muscle Treg suppresses CD4^+^ effector T cells and decreases IFN-γ production to reduce the severity of muscle dystrophy. IL-5 cytokine promotes eosinophil production and is essential for the development of type II inflammation in injured muscle. This cytokine decreases in Treg depletion experiments in *mdx* mice [113]. Tregs may regulate the eosinophil-mediated inflammatory response via IL-5, causing M2 macrophage activation, and delaying Duchenne muscular dystrophy progression, but this is unproven. The inhibitory effect of Tregs on muscle inflammation is dependent on the biology of the immunomodulatory cytokine IL-10, with IL-10 deficiency significantly exacerbating muscle dystrophy in *mdx* mice [114]. H3 relaxin anti-fibrotic factor is overexpressed in *mdx* mice and is produced by Tregs [115]. This factor may act synergistically with IL-10 to inhibit muscle inflammation. Interestingly, acute or partial depletion of Tregs failed to induce differential IL-10 expression, suggesting a possible compensatory regulatory mechanism of IL-10. Further exploration of IL-10 regulatory mechanisms and the functional role of Treg-derived IL-10 in the pathogenesis of myotonic dystrophy is required. Transfer of Tcra and Tcrb recombinant genes (TCR transgenes) into an *mdx* mouse model induces muscle Treg aggregation and improves muscle repair and regeneration showing that this may be a transgenic treatment method of muscular dystrophy [36]. ATP released from necrotic muscle fibers and inflammatory cells inhibits Tregs by activating purinergic P2X receptors. Blocking the ATP/P2X purinergic signalling pathway induces an increase in Tregs in the *mdx* mouse model while attenuating the inflammatory response disease to delay the progression of muscular dystrophy [116]. Recently, dystrophin-specific T cell responses were observed in a subset of patients with myotonic dystrophy treated with Treg-related gene therapy alleviating inflammation and suppressing the immune response [117, 118]. In the future, Treg-associated gene therapy is likely to be combined with immunotherapy and has great potential in the treatment of dystrophy (Fig. 4). However, more studies are needed to demonstrate the upstream and downstream regulatory mechanisms of Treg in Duchenne muscular dystrophy.

## Conclusion and perspective

Due to the heterogeneity and high plasticity of Treg cells, different Treg subpopulations and different sites of Treg are unique in terms of transcription factor expression, chemokine and adhesion receptor recruitment, and effector functions [15]. Most studies assessing the regenerative role of Tregs have focused on the traditional CD4+ CD25+ FoxP3+ Treg population; however, there is controversy regarding the source of Tregs that exert regenerative effects after skeletal muscle injury. Only i-Tregs, not n-Tregs obtained from the spleen enhance muscle stem cell kinetics after co-culturing experiments, suggesting that i-Tregs play an important role in muscle repair and regeneration [50]. However, n-Tregs, not i-Tregs mainly appear after muscle injury with a specialized Treg subset accumulating in muscle to play a reparative role in damaged muscle [8]. Therefore, the findings derived from in vitro combined Treg culture of MuSCs are not specific enough to draw strong conclusions and require further research [7]. In addition, under certain conditions, Th cells can be transformed into Treg. By constructing a non-obese diabetes mouse model, You et al. [51] showed that the Treg subpopulation detected in the spleen was not derived from the expansion of thymic Treg but from the transformation of other cells. Later, Laurence et al. [52] further illustrated in a graft-versus-host reaction mouse model that signal transducer and activator of transcription-deficient initial Th cells may tend to transform into Treg cells. In addition, some Treg cell subsets that do not express FoxP3 (e.g., CD8+ CD122+ Treg, CD4+ Tr1 and Th3Treg, CD4-CD8-αβT cells, γδT cells, CD8+ CD28-T cells) have also been found to have this regulatory function; for example, CD8+ CD28-T cells can suppress CD4+ T cell activation and proliferation by releasing IL-10 and TGF-β [90, 119–121]. In conclusion, the origin of the part of treg involved in muscle damage repair deserves to be explored, which is crucial to guide the treatment of skeletal muscle-related diseases. Most current studies consider Tregs as an important player in muscle repair. Fibro/adipogenic progenitor cells in the muscle sense the signal of the injury stimulus and release alarmin IL-33 which activates and recruits a large number of Tregs to the site of injury through the action of the IL-33:ST2 protein axis. Tregs stimulate muscle stem cell differentiation and muscle repair and regeneration by promoting the polarization of M1 pro-inflammatory phenotype macrophages to M2 anti-inflammatory phenotype macrophages and the release of the specific growth factor dual regulator Areg. Although the IL-33:ST2 protein axis is thought to be critical for Treg aggregation after injury, lack of IL-33 does not cause immune disease in humans indicating that the pro-regenerative effects of IL-33-regulated Treg may differ between mice and humans [122]. Further studies are needed to elucidate the mechanisms and any pathways regulating the massive aggregation of Treg in damaged skeletal muscle to provide new therapeutic targets for Treg-induced regenerative effects in skeletal muscle for clinical purposes. In addition, Tregs may play a role in suppressing IFN-γ production by effector T cells, and activating CIITA transcription factor leading to upregulation of MHCII expression in macrophages. The mechanism by which Tregs regulate effector T cells in muscle to affect skeletal muscle is still unknown. Specific knockdown or overexpression of Areg gene in skeletal muscle injury models are necessary to determine the function of Treg secretion of Areg to exert muscle repair. Furthermore, it is unclear how Tregs can play such a large role in tissue repair and regeneration given that they represent only a small fraction of the total number of immune cells. Further investigation is needed to determine whether Tregs influence the repair process by regulating other immune cells in addition to mediating tissue repair and regeneration through macrophage needs.

Targeted chimeric antigen receptor-Treg cell therapy is an emerging therapeutic tool with broad therapeutic prospects for tumours, transplantation, and autoimmune diseases. Preclinical animal models for T1 diabetes, autoimmune hepatitis, inflammatory bowel disease, encephalitis, arthritis, and other diseases are successfully established [123–125]. However, its application in skeletal muscle injury repair is unexplored and its ability to play a role in skeletal muscle injury needs to be further explored. Targeted chimeric antigen receptor-Treg cells or drugs that can amplify Treg cells in vivo could be used in the future as a potential treatment for muscular dystrophy in combination with gene therapy, with the main advantage that they may not produce the same severe side effects as glucocorticoid therapy; the immunosuppressive ability of Treg cells to suppress muscular dystrophy during gene therapy still needs to be studied in depth. Secondly, Treg cells can be used as a potential therapeutic target for mdx muscular dystrophy, and the progression of the muscular dystrophy phenotype can be delayed by accumulating Treg cells in the muscles of dystrophic mdx mice through therapeutic approaches such as injecting bone bridge protein intervention, blocking the extracellular ATP/P2X purinergic signalling pathway, and injecting IL-2c and Areg [126]. In addition, the regulatory role of innate immune cells (e.g., DC) on Treg cells cannot be ignored. For example, they can promote the proliferation of Treg cells, DC can induce the differentiation of naive CD4+ T cells to Treg cells and influence the migration of Treg to peripheral lymphoid organs and surrounding tissues; the exploration of these mechanisms is beneficial to promote the accumulation of local Treg cells in skeletal muscle, which is essential for its injury repair.

Although there is substantial evidence in animal experiments and in vitro cellular assays that Tregs promotes skeletal muscle repair and regeneration, the results may not be reproducible in human experiments due to the complexity of the human immune system. The unknown cytokine interaction effect in humans is one of the predictable side effect risks, with other unpredictable side effects amongst the first challenges that need to be overcome during Treg application for human skeletal muscle injury treatment. Currently, two markers are used to differentiate and isolate human Tregs; Helios only distinguishes 70% of human Tregs due to the influence of Foxp3 expression [127], while low expression of IL-7 receptor CD127 is negatively correlated with the number of cells in human pTreg. CD127-isolated Tregs have the same inhibitory activity as tTregs suggesting that CD127 may be a suitable marker for Treg isolation [128]. In conclusion, unpredictable side effects and limitations in the isolation and identification of human Tregs and in vitro amplification techniques suggest that targeted chimeric antigen receptor-Treg therapy requires further experimental studies before use in the clinical field for human skeletal muscle repair.

## Supplementary information

checklist

## Data Availability

All the data supporting the findings of this study are available from the corresponding author on reasonable request.
